# Polarization-Independent Large Third-Order-Nonlinearity of Orthogonal Nanoantennas Coupled to an Epsilon-Near-Zero Material

**DOI:** 10.3390/nano11123424

**Published:** 2021-12-17

**Authors:** Wenjuan Shi, Hongjun Liu, Zhaolu Wang

**Affiliations:** 1 State Key Laboratory of Transient Optics and Photonics, Xi’an Institute of Optics and Precision Mechanics, Chinese Academy of Sciences, Xi’an 710119, China; shiwenjuan@opt.cn (W.S.); wangzhaolu@opt.ac.cn (Z.W.); 2 University of Chinese Academy of Sciences, Beijing 100084, China; 3 Collaborative Innovation Center of Extreme Optics, Shanxi University, Taiyuan 030006, China

**Keywords:** orthogonal nanoantennas, epsilon-near-zero material, nonlinear refractive index, polarization-independent

## Abstract

The nonlinear optical response of common materials is limited by bandwidth and energy consumption, which impedes practical application in all-optical signal processing, light detection, harmonic generation, etc. Additionally, the nonlinear performance is typically sensitive to polarization. To circumvent this constraint, we propose that orthogonal nanoantennas coupled to Al-doped zinc oxide (AZO) epsilon-near-zero (ENZ) material show a broadband (~1000 nm bandwidth) large optical nonlinearity simultaneously for two orthogonal polarization states. The absolute maximum value of the nonlinear refractive index n_2_ is 7.65 cm^2^∙GW^−^^1^, which is 4 orders of magnitude larger than that of the bare AZO film and 7 orders of magnitude larger than that of silica. The coupled structure not only realizes polarization independence and strong nonlinearity, but also allows the sign of the nonlinear response to be flexibly tailored. It provides a promising platform for the realization of ultracompact, low-power, and highly nonlinear all-optical devices on the nanoscale.

## 1. Introduction

The nonlinear optical response of common materials is limited by a large device size, high energy consumption, narrow bandwidth, and polarization sensitivity, which seriously affect their applications in all-optical signal processing, harmonic generation, quantum information processing, etc. Therefore, strong nonlinear optical materials are needed to overcome these limitations and realize high-performance all-optical devices.

Epsilon-near-zero (ENZ) materials are a new kind of promising nonlinear optical media. ENZ materials exhibit a vanishing real part of permittivity in certain spectral ranges and strong localization of the field, which is a class of zero-refractive-index (NZI) materials [1,2,3,4,5] with a high nonlinear coefficient [6,7] and unprecedented ultrafast nonlinearity in the subwavelength propagation length. Al-doped zinc oxide (AZO) is an ideal ENZ material; it has a lower loss [8] and confines light better than indium tin oxide (ITO). However, the ENZ material only has a large nonlinear response in a relatively narrow spectral range and is sensitive to polarization, which limits its application.

At present, metamaterials, meta-surfaces [9,10,11,12], plasmonic structures [13,14], and composite nanostructures [15,16,17] have been explored to enhance the optical field and the optical nonlinear response of materials. However, the nonlinearity generated in these structures has a balance of strength and peak position, which can be overcome by designing nanostructures on ENZ materials. Recently, Alam et al. [18] proposed nanoantennas coupled to an epsilon-near-zero material; because of the strong nonlinearity of EZN materials and strong coupling of nanoantenna resonances, a larger nonlinearity can be achieved. Furthermore, the sign and amplitude of the refractive index are well controlled by wavelength, but it is very sensitive to polarization. Strong nonlinearity occurs only when the polarization direction of the incident light is parallel to the long axis of the antenna. Niu et al. [19] proposed a polarization-selected nonlinearity transition in gold dolmens coupled to an epsilon-near-zero material and realized the change of the sign of nonlinear coefficients through polarization conversion. In addition to studying metal nanoantennas, dielectric nanoantennas have also gained increasing interest due to lower loss. Davide Rocco et al. [20] proposed a boosting second harmonic radiation from AlGaAs nanoantennas with epsilon-near-zero materials. D. Rocco et al. [21] analyzed the impact of losses on second-order nonlinear processes and found that the overall conversion efficiency strongly depends on the damping of the substrate rather than the optimization of the resonator. However, these studies are all based on ITO and CdO films, and there are few studies on the strong nonlinearity of the coupling structure based on AZO materials. The AZO material is nontoxic, cheaper and has a lower loss than ITO, so the study of nonlinearity based on the AZO material provides an important reference value for choosing better strong nonlinear materials.

In this paper, we propose a center-symmetric orthogonal nanoantenna array on an AZO film. The nanoantennas generate a strong plasmon mode, i.e., localized electric field enhancements, and overcome the impedance mismatch. It has stronger nonlinear and polarization insensitivity at normal incidence benefiting from strong coupling. The absolute maximum value of the nonlinear refractive index of the two polarization states is 7.65 cm^2^∙GW^−^^1^ at I = 150 MW∙cm^−^^2^, which is 4 orders of magnitude higher than that of the bare AZO thin film [7] and 3 orders of magnitude larger than that of the highly nonlinear metamaterial and the bare ITO film [6,22]. The effective third-order nonlinear susceptibility also provides a greater than 210000-fold enhancement with polarization insensitivity, which is 9.1 times larger than the literature [19].

## 2. Materials and Methods

**Figure 1:**
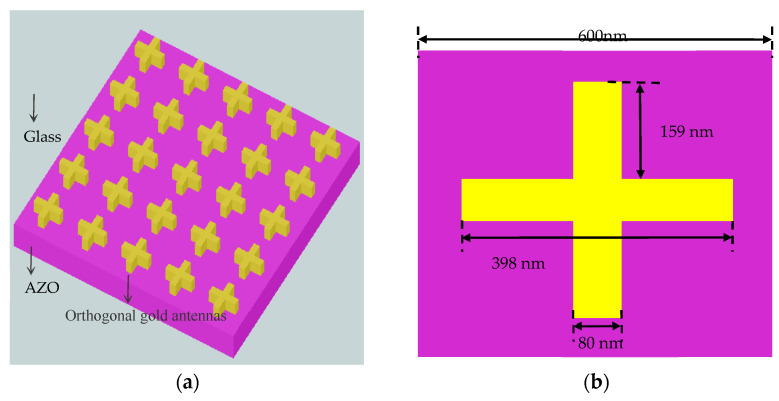
Structure (**a**) The structure shows—from top to bottom—gold orthogonal nanoantennas with height 25 nm, AZO nanolayer with height 22.5 nm, glass (**b**) Planar structure of the gold orthogonal nanoantennas, the distance (600 nm) of the two adjacent orthogonal antennas.

The structure of the polarization-independent strong nonlinearity is a center-symmetric orthogonal nanoantenna array on the AZO film, and there is no air gap between the nanoantennas and the substrate, as shown in Figure 1. The AZO film supports the ENZ mode at thin enough, has a large density of states and can enhance the interaction of light-matter. The thickness of the ENZ material is generally selected to be ≤1/50 the wavelength of the ENZ ($\lambda_{ENZ}$) [23]. The size of the array on a glass substrate is 500 × 500 μm^2^, with a distance (600 nm) of the two adjacent orthogonal antennas. A 22.5-nm-thick AZO layer is sandwiched between the antenna array and the glass substrate, the dimensions of the dipole antennas is 398 nm × 80 nm × 25 nm and the antenna edges are sharp. The position of the plasmon resonance can be adjusted by changing the length of the dipole antenna, utilizing the Finite Different Time Domain (FDTD) method simulation software, ANSYS Lumerical 2020, (the source shape is a plane wave, mesh type is auto nonuniform, mesh accuracy is 8, boundary conditions are periodic in x and y directions, boundary condition is perfectly matched layer (PML) in z direction) when the surface plasmon resonance of the dipole antenna is close to the resonance position of the ENZ mode, strong coupling can be achieved.

The real and imaginary parts of permittivity (ε) of the AZO film can be calculated using the Drude model [6,24,25,26,27,28]:

(1) $$\varepsilon =\varepsilon_{\infty }-\frac{\omega_{p}^{2}}{\omega^{2}+i\gamma \omega }$$

(2) $$\omega_{p}^{2}=\frac{ne^{2}}{m_{e}\varepsilon_{0}}$$

(3) $$\gamma =\frac{e}{\mu m_{e}}$$

**Figure 2:**
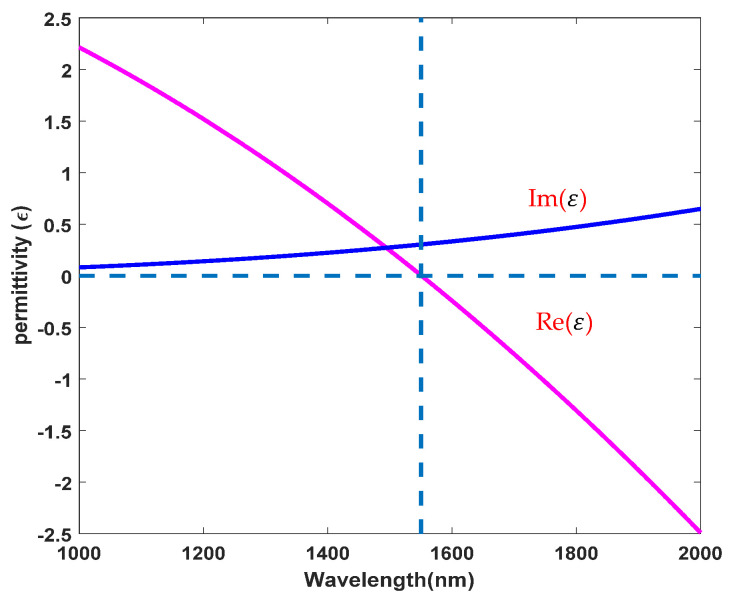
Calculated real and imaginary parts of the permittivity of the ITO film as a function of wavelength.

The parameters of the AZO film are derived from the literature [8], where$\;\varepsilon_{\infty }=3.8$ is the high-frequency permittivity, γ= 9.71 × 10^15^ rad∙s^−^^1^ is the Drude damping rate, $\omega_{p}$= 2.3765 × 10^15^ rad∙s^−^^1^ is the plasma frequency, *ω* is the angular frequency of the incident light, n is the carrier concentration, e=1.602 × 10^−19^ C is the electron charge, $\varepsilon_{0}$ is the permittivity of free space, *μ* is the electron mobility, $m_{e}=0.38m$ is the effective electron mass [29] and m is the electron mass. As shown in Figure 2, it is obvious that the real Re(ε) of the permittivity of the ENZ wavelength ($\lambda_{ENZ}\;$= 1550 nm) vanishes and the imaginary Im(ε) is 0.3.

## 3. Results and Discussions

### 3.1. Linear Characteristic Analysis

**Figure 3:**
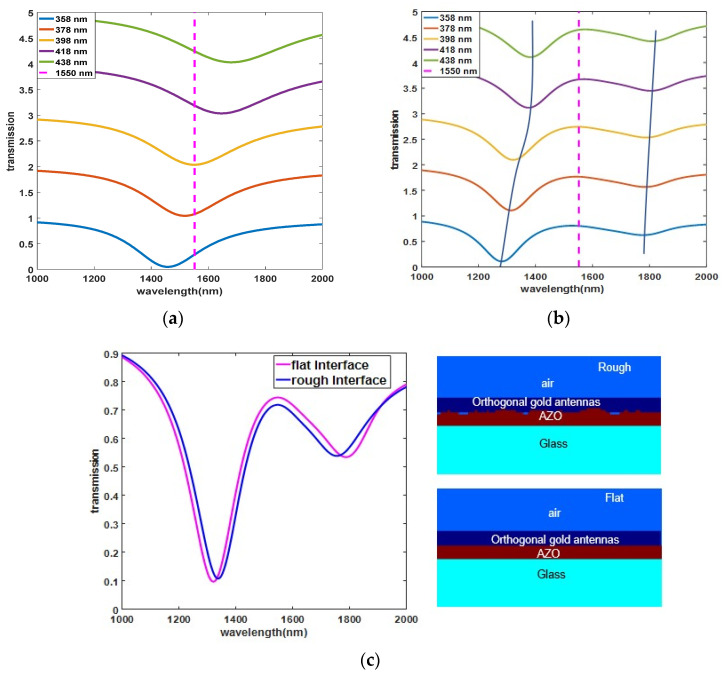
Transmission spectra of x and y polarization. (**a**) Without AZO film; (**b**) With AZO film; (**c**) Transmission spectra for flat interface and rough interface, right-side inset (refractive index monitor views from Lumerical).

Transmission spectra are calculated by the finite difference time-domain (FDTD) method with Lumerical 2020. To determine the optimal length of the orthogonal nanoantennas, the transmission spectra of the structure without the AZO film at normal incidence are calculated, as shown in Figure 3a. The wavelength of the plasmon resonance redshifts as the length of the orthogonal nanoantennas increases. The vertical line in Figure 3a is the $\lambda_{ENZ}$, the wavelength of the plasmon resonance is close to the ENZ wavelength at a length of 398 nm, and the transmission dip appears at *λ* = 1550 nm. Figure 3b shows the transmission spectra with the AZO film at normal incidence. The AZO film supports the eigenmode at $\lambda_{ENZ}$, exhibits a large density of states and homogeneously confines electromagnetic radiation within the film [30,31,32]. The antishift between the plasmonic mode and the ENZ mode indicates strong coupling-induced Rabi splitting. The linear transmission spectra of the coupled structure show two distinct resonances that result from strong coupling; the main resonance wavelength is *λ* = 1346 nm and the weaker resonance wavelength is *λ* = 1879 nm. The wavelength separation between the two resonances (~533 nm) is larger than the 3 dB linewidth of the orthogonal nanoantenna resonance on a glass substrate alone [33,34], which is beneficial for realizing wide-band strong nonlinearity. The interface plays an important role in the real fabrication of metamaterials [35,36], so the effect of interface roughness on linear and nonlinear properties should be considered in practical applications. We compare the transmission spectra of rough and flat AZO interfaces in the coupled structure, and the results are shown in Figure 3c: (1) the main resonance is redshifted, and the weak resonance is blueshifted in the rough interface. (2) The difference between the two resonance wavelength (~447 nm) of the rough AZO interface is lower than that of the flat AZO surface. The influence of interface roughness on the linear transmission spectrum is relatively weak in this structure. In addition, the linear characteristics are consistent with polarization independence for the x and y polarizations.

### 3.2. Field Distributions and Enhancement

**Figure 4:**
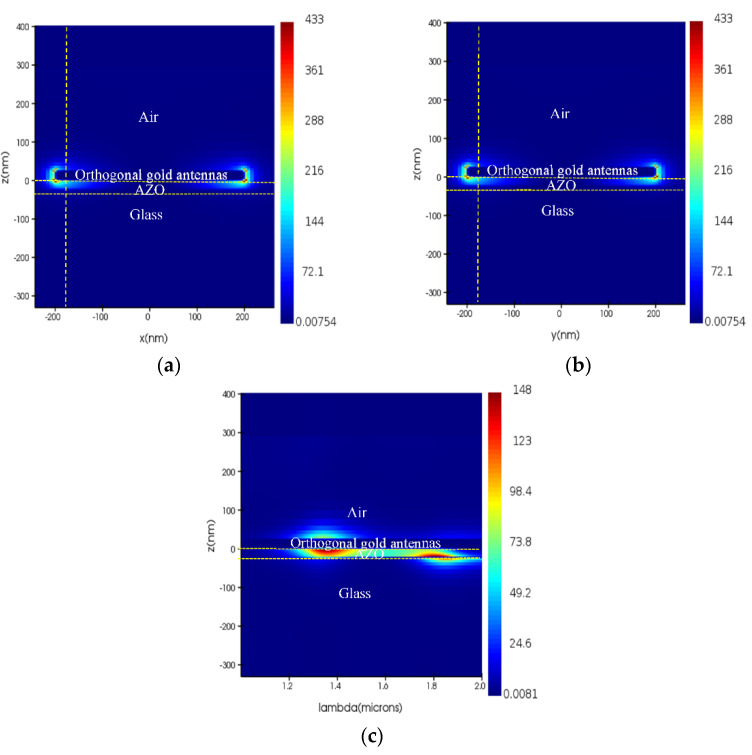
Electric field distribution and intensity enhancement of the resonance at a wavelength 1346 nm. (**a**) X polarization; (**b**) Y polarization; (**c**) Wavelength-dependent intensity enhancement.

Another way to enhance the nonlinear effect is to enhance the strong coupling of the plasmon mode and the ENZ mode. The strong coupling, which greatly increases the electric field strength of the ENZ film, effectively confines the light in the ENZ film and overcomes the obstacle of impedance mismatch at normal incidence. Figure 4a,b shows the normalized field intensity ${\left|E\right|}^{2}/{\left|E_{0}\right|}^{2}$ distribution of the resonance at the wavelength of 1346 nm in the x and y polarization, respectively. The field intensity distribution shows polarization independence and is mainly distributed to both ends of the orthogonal antenna. To avoid the hot-spot effect, we analyze the relationship between the field intensity enhancement and the wavelength at the position of the white line in Figure 4a,b. The normalized field intensity ${\left|E\right|}^{2}/{\left|E_{0}\right|}^{2}$ is increased by 148 times in the AZO film as shown in Figure 4c, which is beneficial for enhancing the optical nonlinear response.

### 3.3. Nonlinear Response Enhancement Mechanism

According to the continuity of the normal component of the electric displacement field, that is, $\left|E_{⊥}\right|\propto \varepsilon^{-1}\left|E_{0,⊥}\right|$ [5], (where $E_{⊥}$ is the normal electric field in the ENZ medium, and $E_{0,⊥}$ is the external incident electric field), ENZ materials have a strong field enhancement due to the small magnitude of the permittivity (ε). The strong coupling of the ENZ orthogonal nanoantenna structure provides a larger optical field enhancement than that of the bare AZO film and reduces the intensity threshold to achieve a nonlinear response. Owing to the strong coupling of plasmonic-ENZ, the resonance wavelength of the structure is very sensitive to the ε of the substrate materials and structural parameters. The refractive index change is ∆n = $∆\varepsilon /2\sqrt{\varepsilon }$ for lossless materials. Therefore, when the pump light is incident, a small change (∆ε) in permittivity of the AZO film will cause the resonant wavelength of the structure to shift and cause a larger ∆n. In addition, due to the change in spectral position when the pump light is incident, the sign of ∆n can be changed by the wavelength. By choosing appropriate orthogonal nanoantenna parameters and ENZ substrates, the sign and magnitude of the nonlinear coefficient can be well controlled to meet actual needs [6,7].

We use a two-temperature model [37] to study the ultrafast optical nonlinear response mechanism of AZO films. It has been successfully used to explain the ultrafast nonlinear response of femtosecond pulsed laser radiation in metals [38,39,40]. The response is described by [33]:

(4) $$C_{e}\frac{\partial T_{e}}{\partial t}=-g_{ep}\left(T_{e}-T_{l}\right)+\frac{N}{2\tau_{ee}}$$

(5) $$C_{l}\frac{\partial T_{l}}{\partial t}=g_{ep}\left(T_{e}-T_{l}\right)+\frac{N}{2\tau_{ep}}$$

(6) $$\frac{\partial N}{\partial t}=-\frac{N}{2\tau_{ee}}-\frac{N}{2\tau_{ep}}+p$$

where $T_{e}\;(T_{l})$ is the free-electron (lattice) temperature, $g_{ep}$ = 1.4 × 10^16^ Wm^−3^∙K^−1^ is the electron-phonon coupling coefficient [41], $C_{e}$ = 3500 Jm^−3^∙K is the heat capacity of the electrons [41],$\;C_{l}$ = 2.83 × 10^6^ Jm^−3^∙K is the heat capacity of the lattice, N is the nonthermal energy density stored in the excited electrons [42], $\tau_{ee}$ $\left(\tau_{ep}\right)$ is the electron-electron (electron-phonon) relaxation time [43,44], and *P* is the time-dependent absorbed power density:

(7) $$p=\left(1-R-T\right)I_{0}\alpha exp\left[-2{\left(\frac{t}{\tau_{p}}\right)}^{2}\right]$$

where *R*, *T*, and *α* are the reflectance, absorptivity, and absorption coefficient, respectively, and $\tau_{p}$ is the laser pulse relaxation time, and $I_{0}$ the is intensity of the incident light.

**Figure 5:**
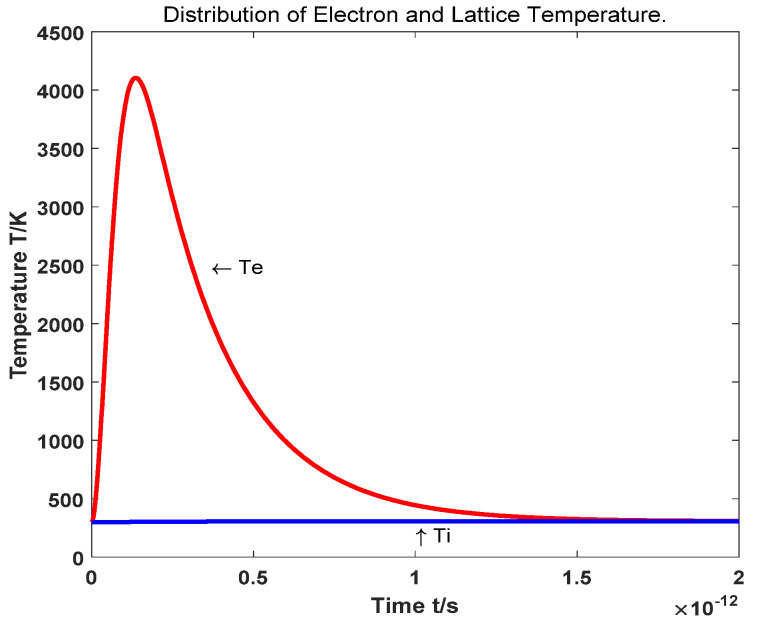
The function of electron temperature and response time.

The maximum electron temperature at normal incidence can be calculated by solving the two-temperature model. The plasma frequency of the AZO film at high electron temperature can be obtained by Formula (6). Figure 5 shows the function of electron temperature and response time:

(8) $$\omega_{p}^{2}\left(T_{e}\right)=\frac{e^{2}}{3m\varepsilon_{0}\pi^{2}}\int_{0}^{\infty }dE{\left(1+2CE\right)}^{-1}{\left(\frac{2m}{\hbar^{2}}\left(E+CE^{2}\right)\right)}^{\frac{3}{2}}\left(-\frac{\partial f_{FD}\left(E,\mu \left(T_{e}\right),T_{e}\right)}{\partial E}\right)$$

where C=0.33 eV^−1^ is the nonparabolicity parameter of the thin AZO film [45]. ℏ is the reduced Planck constant, and $-\frac{\partial f_{FD}\left(E,\mu \left(T_{e}\right),T_{e}\right)}{\partial E}$ is a measure of the thermal broadening intensity of the electron distribution around the Fermi level.

### 3.4. Nonlinear Coefficient

**Figure 6:**
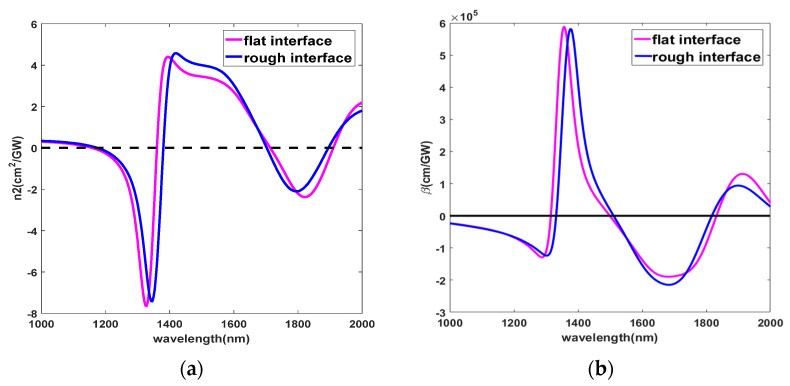
Nonlinear coefficient of flat and rough interfaces: (**a**) Effective nonlinear refractive index $n_{2}$ in x and y polarization, (**b**) Nonlinear absorption coefficient β in x and y polarization.

The effective refractive index of the coupled structure is calculated by the S parameter in the FDTD simulation [46], and pulse width of 150 fs and I = 150 MW∙cm^−2^ are used in the simulation. The wavelength-dependent effective nonlinear index coefficient $n_{2}$ and absorption coefficient β can be calculated by $n_{2}=Re\left(∆n/I\right)$ and $\beta =\frac{4\pi }{\lambda }$[Im(∆n/I)], ∆n is the difference in refractive index of the coupled structure with or without pump light intensity, and I is the pump light intensity. Figure 6a,b show the functions of $n_{2}$ and β with wavelengths in flat and rough interfaces, respectively. The structure exhibits polarization insensitivity, and the magnitude and sign of the nonlinear coefficient can be controlled by the wavelength. In the flat interface, $n_{2}$ is negative and shows self-defocusing in the wavelength range of 1158 nm≤λ≤1357 nm and 1717 nm≤λ≤1908 nm, $n_{2}$ is positive in the rest of the wavelengths and shows self-focusing, as shown in Figure 6a. The minimum and maximum value of $n_{2}$ at λ = 1328 nm and 1390 nm are related to the main resonance, the maximum of $\left|n_{2}\right|$ is 7.65 cm^2^∙GW^−1^. The nonlinear refractive index at λ = 1300 nm is not only 20,000 times larger than that of the bare AZO film [7] but also 3 orders of magnitude larger than that of the highly nonlinear metamaterials and the bare ITO film [6,20]. Additionally, it is 7 orders of magnitude larger than the glass and 4 orders of magnitude larger than chalcogenides in a similar spectral range [47]. The nonlinear optical enhancement bandwidth is in the range of 1000 nm ≤ λ ≤ 2000 nm, and $\left|n_{2}\right|\geq 1$ cm^2^∙GW^−1^ accounts for 61% of the total wavelength. In the rough interface, $n_{2}$ is negative in the wavelength range of 1177 nm≤λ≤1379 nm and 1705 nm≤λ≤1893 nm, the minimum and maximum value of $n_{2}$ at λ = 1346 nm and 1418 nm are related to the main resonance, the maximum of $\left|n_{2}\right|$ is 7.42 cm^2^∙GW^−1^. Although $\left|n_{2}\right|$ is slightly lower than that of the flat interface, the bandwidth of nonlinear enhancement is similar to the flat interface. 

Similarly, the magnitude and sign of the nonlinear absorption coefficient also depend on the position of the resonance wavelength, as shown in Figure 6b. In the flat interface, the nonlinear absorption coefficient *β* is a negative value in the wavelength range of λ≤1314 nm and 1500 nm≤λ≤1831 nm and exhibits saturated absorption; *β* is positive in the rest of the wavelengths and shows anti-saturated absorption, the maximum *β* value is 5.88 × 10^5^ cm∙GW^−1^. In the rough interface, *β* is a negative value in the wavelength range of λ≤1332 nm and 1509 nm≤λ≤1818 nm and the maximum *β* value is 5.794 × 10^5^ cm∙GW^−1^. Therefore, the structure we propose in this study can control the magnitude and sign of the nonlinear coefficient by the wavelength, and realize polarization insensitivity. The rough interface has little influence on the nonlinear coefficient.

### 3.5. Enhancement of Third-Order Susceptibility

The electric field strength of the third-order polarization $P^{\left(3\right)}$ depends on the strength of the incident electric field in the nonlinear response [47]:

(9) $$P^{\left(3\right)}=\varepsilon_{0}\chi^{\left(3\right)}E^{3}$$

where $\chi^{\left(3\right)}$ is the third-order susceptibility,$\;\chi^{\left(3\right)}$ = 3.5 × 10^−19^ m^2^∙V^−2^ of the AZO film [48], and $\chi^{\left(3\right)}$ = 7.56 × 10^−19^ m^2^∙V^−2^ of gold.

**Figure 7:**
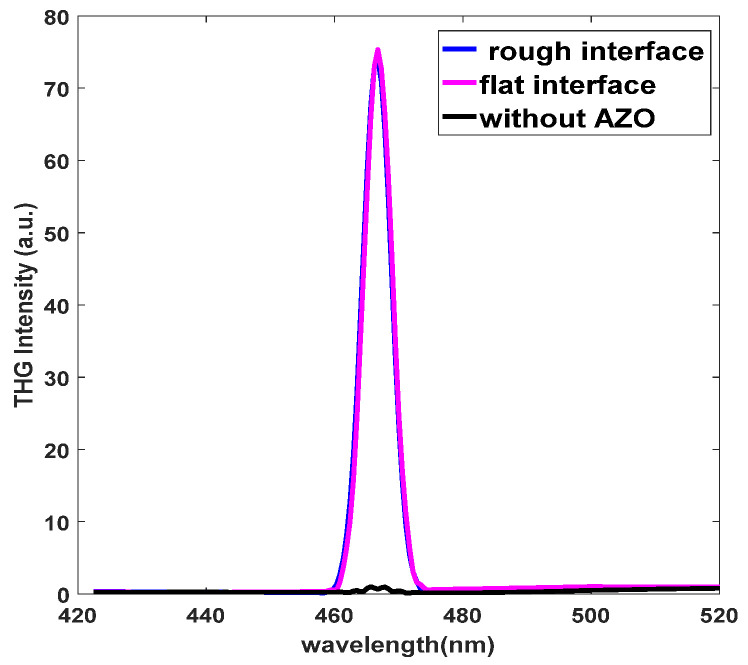
Transmission spectrum of THG in x and y polarization.

The $\chi^{\left(3\right)}$ of the coupled structure also shows broadband enhancement and is polarization-independent. It can be measured by the third harmonic generation (THG), under the same conditions (normal incidence of the x and y polarization) and an excitation wavelength of 1400 nm, the simulated THG nonlinear transmission spectrum in the coupled structure and the structure without the AZO film are shown in Figure 7. The THG signal peak is observed at 466.67 nm in the coupled structure, but there is no obvious transmission peak on the structure without the AZO film, which illustrates that the THG signal is mainly generated from the AZO film, and the orthogonal nanoantennas mainly enhance the electric field strength by strong coupling. In addition, Figure 7 also shows the THG transmission spectrum of the rough interface, which is slightly lower than that of the flat interface.

**Figure 8:**
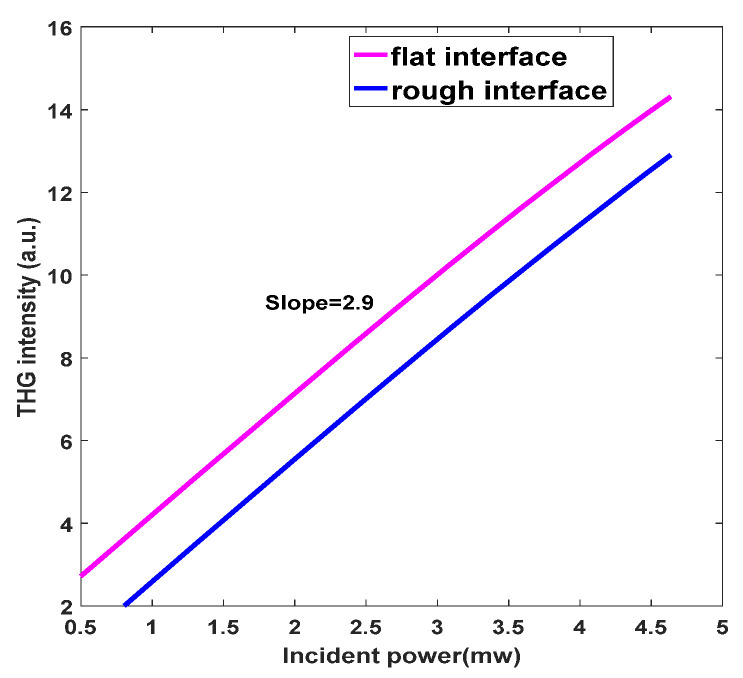
The function of pump power and THG power.

In the simulation, $P_{\omega }=I_{\omega }S_{unit}$, $I=\frac{1}{2}Re\left(E\times H^{*}\right)$, $I_{\omega }$ is the peak intensity of the pump light, $S_{unit}$ is the area of the structural unit cell, *E* is the pump electric field, and $H^{*}$ is the conjugation of the magnetic field. The polarization intensity can be changed by changing the pump intensity. Figure 8 is a function of pump power and THG power in the flat and rough interfaces, and the x and y coordinates are taken logarithmically. The slope of the pump power and the THG power is 2.9 for both the flat and rough interfaces, which is close to the theoretical value of 3. This illustrates that the signal is truly generated by the THG conversion, which is dominated by $\chi^{\left(3\right)}$ of the nonlinear response.

**Figure 9:**
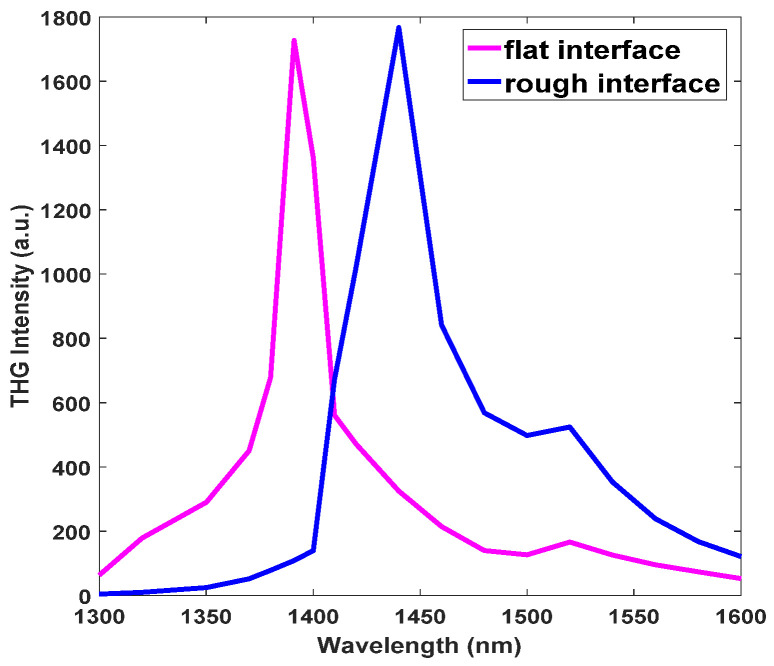
The function of the THG signal and excitation wavelength of the coupled structure.

To analyze the broadband enhancement of $\chi^{\left(3\right)}$ in the structure, the THG signal in the excitation wavelength range of 1300–1600 nm in the coupled structure is analyzed. The orthogonal nanoantennas structure has realized strong coupling, overcomes the obstacles of incident angle and impedance mismatch, and reduces the demand for intensity [49]. Figure 9 shows the function of the fundamental wave and the THG signal in the flat and rough interfaces. In the flat interface, the THG signal is the largest at a wavelength of 1390 nm, and the maximum enhancement factor compared with the bare AZO film is 210,000, which is 9.1 times larger than that in the literature [19]. In the rough interface, the THG signal is the largest at a wavelength of 1440 nm and is redshifted compared to the flat interface, the maximum enhancement factor is similar to the flat interface. In addition, in the flat interface, the intensity of the THG signal caused by strong coupling can be enhanced three orders of magnitude in the bandwidth of 300 nm (1300–1600 nm). The intensity of the THG signal is proportional to $\chi^{\left(3\right)}$, which indicates that $\chi^{\left(3\right)}$ can be increased by three orders of magnitude in the broadband range by comparing the enhancement of the THG intensity of the coupled structure and bare AZO film.

**Figure 10:**
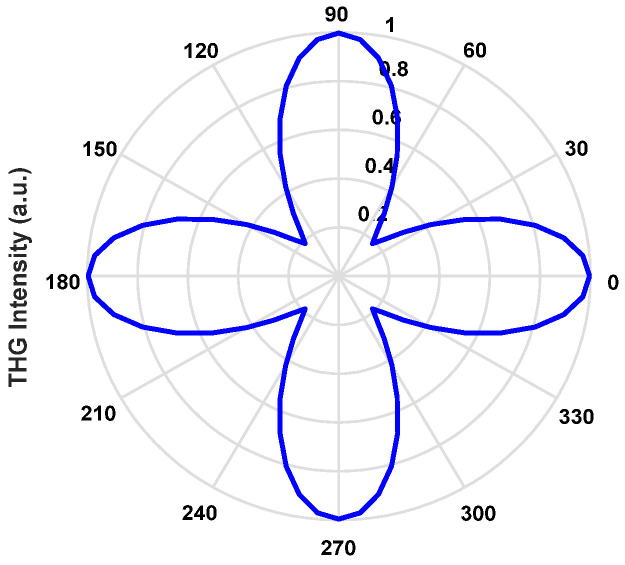
The function of the polarization direction and the intensity of THG in the flat interface.

In addition, we have also achieved polarization insensitivity in a symmetrical structure, as shown in Figure 10. The THG signal can be achieved in the range of polarization angles of 0–360°, and the maximum of THG is on the x and y-polarizations. In summary, the coupled structure achieves polarization insensitivity, a wide band, and strong nonlinearity and solves the limitations of large device size, high energy consumption, narrow bandwidth, and polarization sensitivity in all-optical signal processing and on-chip integrated devices.

## 4. Conclusions

We propose a strongly coupled structure composed of orthogonal nanoantennas on an AZO film to achieve a strong optical nonlinear polarization-independent response. The absolute maximum value of the nonlinear refractive index $n_{2}$ is 7.65 cm^2^∙GW^−1^ with two polarization at normal incidence, and the nonlinear coefficient is enhanced by 4 orders of magnitude compared with the bare AZO film and 7 orders of magnitude compared with silica. The nonlinear refractive index can be enhanced over the entire bandwidth of 1000 nm. In addition, the transition from self-focusing to self-defocusing and saturated absorption to anti-saturated absorption can be realized by controlling the wavelength. The $\chi^{\left(3\right)}$ of the two polarization states is also enhanced three orders of magnitude in the broadband range. Therefore, this coupled structure realizes a broadband, polarization-insensitive strong nonlinear response, and the magnitude and sign of the nonlinear coefficient can be controlled by reasonable selection of nanoantennas parameters. This structure overcomes the obstacle of the weak nonlinear response of common materials and provides a promising platform to design nanocompact and on-chip integrated, polarization-independent strong nonlinear optical devices. 

## Data Availability

The data presented in this study supporting the results are available in the main text. Additional data are available upon request to the corresponding author.
